# No evidence for the immunocompetence handicap hypothesis in male humans

**DOI:** 10.1038/s41598-018-25694-0

**Published:** 2018-05-09

**Authors:** Judyta Nowak, Bogusław Pawłowski, Barbara Borkowska, Daria Augustyniak, Zuzanna Drulis-Kawa

**Affiliations:** 1 Department of Human Biology, University of Wroclaw, Kuźnicza 35, 50–138 Wrocław, Poland; 2 Institute of Genetics and Microbiology, University of Wroclaw, Przybyszewskiego 63/77, 51–148 Wrocław, Poland

## Abstract

The observations that testosterone might be immunosuppressive, form the basis for the immunocompetence handicap hypothesis (ICHH). According to ICHH only high-quality individuals can maintain high levels of testosterone and afford the physiological cost of hormone-derived immunosuppression. The animal and human studies that attempted to support the ICHH by precisely defined impairment of immunity associated with high testosterone levels are inconclusive. Furthermore, human studies have used only selected immune functions and varying testosterone fractions. This is the first study examining the relationship between multiple innate and adaptive immunity and serum levels of free testosterone, total testosterone, DHT and DHEA in ninety-seven healthy men. Free testosterone and marginally DHT levels were positively correlated with the strength of the influenza post-vaccination response. Total testosterone and DHEA showed no immunomodulatory properties. Our findings did not support ICHH assumptions about immunosuppressive function of androgens. In the affluent society studied here, men with higher levels of free testosterone could afford to invest more in adaptive immunity. Since the hormone-immune relationship is complex and may depend on multiple factors, including access to food resources, androgens should be treated as immunomodulators rather than implicit immunosuppressants.

## Introduction

According to evolutionary hypotheses, the sex hormone-dependent morphological traits controlled by sexual selection might signal an individual’s biological condition. Female preferences for males with the most elaborated sexual ornaments may therefore reflect a preference for indirect benefits that increase genetic *fitness* of offspring (e.g. the ability to limit the parasite load^1^. One important component of biological condition which regulates an individual’s survival is the ability to cope with infectious diseases, and this function depends on immune system effectiveness. It is well-known that steroid hormones control the development of primary and secondary sexual traits and are involved in all reproductive functions. The observations that sex hormones are also capable of modulating immune response formed the basis for the immunocompetence handicap hypothesis (ICHH) assumptions postulated by^2^. They suggested that gonadal androgens (e.g. testosterone) exert immunosupressive effects. In consequence, only individuals with high biological quality, including immunity, can produce and maintain a high level of testosterone and can afford the physiological costs of this hormone-derived systemic immunosuppression. In accordance with ICHH, the level of body masculinization (morphological traits that are under the influence of testosterone) reflects an individual’s biological quality, especially immune system effectiveness^2^. To validate the ICHH assumption, evidence documenting physiological costs of high sex hormones levels (e.g. down-regulation of immune function by androgens) is required. The immunomodulatory role of sex hormones was previously tested in many species, mostly birds, using both indirect measurement of immune system effectiveness, e.g. parasite load^3–5^ or direct immune parameters^6–8^. Whereas several studies have supported the ICHH assumption^7–9^, contrasting results^6,10^ have been also reported.

Although some studies on humans supported immunomodulatory properties of sex hormones^11–15^ the findings of studies on testosterone influences on immune cells are inconclusive. There are several types of studies which document the influence of human male sex hormones on immunity: (1) *In vitro* studies that attempt to measure the function of isolated immune cells after treatment with various testosterone concentrations; (2) *in vivo* experimental human studies that attempt to measure the changes in various immune parameters after testosterone administration; (3) correlational studies measuring the relationship between serum/saliva hormone concentrations and selected immune functions. The results of these studies are difficult to compare and therefore preclude drawing definitive conclusions about the immunosuppressive properties of testosterone. Furthermore, most of the aforementioned reports are also inadequate to forming conclusions regarding the general effects of testosterone on male immunity, which is necessary to confirm the validity of the ICHH assumption. *In vitro* studies concern only selected cell functions tested on isolated immune cells. The experimental studies performed to date were mostly related to selected groups of people with genetic, hormonal, or immunological disorders^16–19^. In contrast, correlational studies, which can reflect the immune-hormone interactions in natural/physiological conditions are still incomplete. Due to the complexity of immune-hormones interactions and possible viability-fecundity trade off affecting an individual’s fitness benefits, the correlational studies might be not a conclusive test of ICHH assumptions. Yet, answering the question about relationship between androgens level and immunity in men gives us deeper insight into the complex interrelationship between those aspects of physiology that are crucial for the hypothesis related to the mechanisms of sexual selection.

The previous correlational studies have mostly focused on a single immune function and one or two androgen fractions. Furthermore, the authors did not control common immunomodulatory factors such as participants’ age, adiposity, or general health status, as measured by basic biomedical indicators (e.g.)^20–22^. It is worth noting that the immune system is a complex network consisting of many mechanisms, organs, cells, receptors, and mediators which cooperate with each other at the molecular level^23^. Therefore, measuring only selected immune parameters does not truly reflect the general immune quality. The human immune system is subdivided into two arms: Innate and adaptive immunity. Innate immunity is composed of antigen-independent mechanisms which mainly constitute the first line of immune defense and inhibit infection until adaptive immunity is developed. Adaptive immunity involves primarily highly specific T and B lymphocytes, which can adapt to the infection and have the ability to “remember” it in antigen-dependent reactions^23^. Additionally, some authors also postulate the trade-off between different immune functions^24^, when one function is highly effective, other functions may be down-regulated. Thus, to define the role of testosterone in immunocompetence, it would be necessary to investigate various immune functions, both innate and adaptive.

Another problem is that male traits that are under sexual selection and may signal immune quality are not only testosterone-dependent. Apart from testosterone, androgens such as dehydroepiandrosterone (DHEA) and dihydrotestosterone (DHT) can also affect sexual ornamentation and possibly immunity. DHT, which is a highly active form of testosterone, binds to androgen receptors (ARs). There is no doubt that DHT plays an important role during the development of secondary male characteristics and spermatogenesis^25^. Furthermore, DHT is also essential for the patterns of facial and body hair growth, which are important components of male physical attractiveness^26,27^. The individuals with the 5-alpha-reductase deficiency (enzyme that converts testosterone to DHT) have normal levels of testosterone but decreased levels of DHT. In the consequence they have ambiguous external genitalia at birth and no facial or body hair as adults^28^, that indicates that the conversion of testosterone to DHT plays a crucial role in the process of masculinization^29^. Furthermore, DHT, has twofold higher affinity to AR than testosterone, so has higher androgenic potency^30^. Since androgen receptors (ARs) are expressed on immune cells, DHT may also affect immune functions^15,31,32^ and therefore should be also taken into account when testing ICHH assumptions. Although animal studies that observed immunosuppressive role of testosterone, suggests AR-independent pathway of this suppression^33^, the role of the AR-mediated pathway in immune modulation was confirmed in studies on androgen receptors knockout mice (ARKO mice)^15^.

To test ICHH we also studied the relationship between DHEA and immunity. Liu *et al*.^34^ showed that DHEA supplementation is associated with the free testosterone increase in middle-aged men. This suggests that DHEA can be also indirectly related to masculinization level. It is interesting that immunomodulatory function of DHEA was studied in the context of aging, (DHEA decreases with age)^35^, but little is known about DHEA influence on immunity in adult and healthy men. It is well known, however, that DHEA is the main steroid precursor of active androgens and the most abundant androgen in a male serum. DHEA can be metabolized into T and DHT in many cells, including muscles or prostate^36^. The effect of DHEA on a male body may be exerted either by direct binding to ARs^37^, or by its metabolites^38^. There is also limited evidence linking DHEA with masculinity-related characteristics e.g. DHEA supplementation decreases fat body mass in men^39^.

The above evidence suggests that to test the hypothesis that men with elaborate sexual ornaments have also higher biological quality, not only testosterone (both free and total) but also DHEA and DHT levels should be tested in relation to the immune system quality.

The aim of this study was to examine the relationship between several androgens (total testosterone [T], free testosterone [fT], DHT, DHEA) and a variety of immune parameters (neutrophil functions, complement activity, lysozyme activity, and a qualitative and quantitative analysis of B and T lymphocytes). Because both innate and adaptive immune cells express androgen receptors and therefore can be affected by androgens^15^ we measured parameters constituting both arms of immunity. The evaluation of immune system quality is based on both qualitative and quantitative tests and includes: (1) quantitative baseline immune parameters (lymphocyte count, immunoglobulin levels), (2) *ex vivo* stimulation test and (3) *in vivo* stimulation. We used a relatively large sample and controlled the most common immunomodulatory factors and biomarkers of general health status, rather than relying only on a single immune biomarker or type of androgen, as had been done in previous studies.

## Results

The descriptive statistics for all analyzed traits are reported in Table 1.

**Table 1:** Descriptive statistics for the analyzed parameters (N = 97).

	Mean	Range	SD
*age (year)*	27.2	18.97–36.7	4.77
*complement activity (µg/ml)*	179.87	47.14–287.53	58.01
*lysozyme activity* ^1^	0.4	0.08–0.6	0.08
*phagocytic uptake* ^2^	174.7	54.30–429.9	64.28
*ROS production* ^3^	9.0	2.47–60.1	9.21
*IgA level (g/L)*	1.9	0.57–7.3	1.10
*IgG level (g/L)*	12.0	4.16–27.0	4.29
*T lymphocyte count (cells/µl)*.	1515.8	538.13–3422.8	536.46
*B lymphocyte count (cells/µl)*.	246.3	45.33–639.9	119.71
*strength of flu post-vaccination response* ^4^	8.2	1.00–64.0	9.73
*strength of tetanus post-vaccination response (N = 35)* ^4^	8.2	0.69–45.7	9.46
*Proliferation rate after Con A stimulation (N = 62)* ^5^	99.8	7.64–324.8	51.83
*Proliferation rate after PWM stimulation (N = 62)* ^5^	36.1	9.15–106.2	18.46
*free testosterone (pg/ml)*	23.7	2.53–52.6	9.91
*total testosterone (ng/ml)*	5.4	1.91–8.4	1.11
*DHEA(ng/ml)*	15.6	2.79–33.9	6.17
*DHT (pg/ml)*	719.0	364.35–1061.8	128.24
*BMI (kg/m*^2^)	23.7	16.87–29.9	2.85
*BF (%)*	20.8	7.20–32.5	4.80

^1^The difference in absorbance value between control samples (bacteria suspension without lysozyme) and test samples (bacteria treated with serum-contained lysozymes).

^2^Mean fluorescence intensity of blood phagocytes after phagocytosis of fluorescently labeled bacteria.

^3^Mean area under the chemiluminescence curve (AUC_CL_) for stimulated test sample divided by AUC_CL_ for control.

^4^Fold increase in antibody titers (for influenza) or antibody levels (for tetanus) before and after vaccination.

^5^Stimulation index − (CPM for stimulated test sample divided by CPM for unstimulated control).

### Inter-correlation between immune parameters

The majority of immune functions did not correlate with each other. Weak correlations were observed between complement activity and IgA levels (R = 0.26; *p* = 0.01); lysozyme activity and IgG levels (R = 0.26;* p* = 0.01); ROS production and CD3 (R = 0.22; *p* = 0.03) and CD3 and post-vaccination response (R = 0.26; *p* = 0.01).The only moderately strong correlation was between CD3 and CD9 (R = 0.6; *p* < 0.001).

### Inter-correlation between androgens

Free testosterone was positively correlated with DHT (R = 0.57; *p* < 0.001), total testosterone (R = 0.39; *p* < 0.001) and DHEA (R = 0.31; *p* = 0.002). Total testosterone was also positively related to DHT (R = 0.45; *p* < 0.001) but not to DHEA (R = 0.01; *p* = 0.95). The positive correlation was also observed between DHT and DHEA (R = 0.41; *p* < 0.001).

### The correlations between immune parameters and controlled factors or androgens

Absolute T lymphocyte count (R = −0.24; *p* = 0.02) and the strength of the influenza post-vaccination response (R = −0.26; *p* = 0.01) were negatively associated with participants’ age. BMI and body fat were not related to the analyzed immune functions. Among the hormonal factors, fT was positively related to the strength of the influenza post-vaccination response (R = 0.34; *p* < 0.001), while DHT was positively associated with ROS production (R = 0.22; *p* = 0.03), IgG serum levels (R = 0.217; *p* = 0.03), and the strength of the influenza post-vaccination response (R = 0.25; *p* = 0.01). Both total testosterone and DHEA were not correlated with any of the immune parameters measured (Table 2).

**Table 2:** Correlations between immune function parameters and participants’ age, adiposity, and hormone levels.

	Age	BMI	%BF	fT	T	DHEA	DHT
complement activity	0.023	0.019	0.131	−0.002	0.193	0.040	0.064
lysozyme activity	−0.103	−0.153	−0.005	0.116	0.006	0.062	0.061
phagocytic uptake	0.010	0.170	−0.015	−0.150	−0.009	−0.004	−0.114
ROS production	0.033	0.118	0.037	0.103	0.050	0.050	0.221*
IgA level	0.189	0.096	0.035	0.051	−0.042	0.004	−0.050
IgG level	−0.148	−0.112	−0.115	0.052	0.118	0.002	0.217*
T lymphocyte count	−0.243*	−0.040	0.077	0.075	−0.023	0.050	0.053
B lymphocyte count	−0.126	0.100	−0.051	−0.078	−0.072	0.072	0.020
strength of flu post-vaccination response	−0.258*	−0.172	0.054	0.343**	0.045	0.097	0.253*
strength of tetanus post-vaccination response (N = 35)	0.209	0.109	0.096	0.271	0.286	0.136	0.186
Proliferation rate after Con A stimulation (N = 62)	−0.204	0.044	0.069	0.169	−0.059	0.046	0.051
Proliferation rate after PWM stimulation (N = 62)	−0.103	0.129	0.092	0.218	−0.078	0.062	0.098

*p < 0.05; **p < 0.001.

### The relationship between innate immune functions and androgens

Because some immune functions depend on age and BMI, we checked the relationship between hormone levels and immune function when age and BMI were controlled. Multiple regression analysis models were constructed separately for each immune factor as a dependent variable and each hormone as an independent variable. Among innate immune parameters, the phagocytic uptake was marginally negatively correlated with fT (*p* = 0.06), while DHT was marginally positively correlated with ROS production (*p* = 0.07) (Table 3). However, the supplemental analyses showed that the relationship between fT and phagocytic uptake or DHT and ROS production became nonsignificant when removing statistical outliers for the respective immune parameters from the models. Total testosterone and DHEA were not associated with any of the immune factors tested (Table 3).

**Table 3:** Multiple regression models showing the relationship between each androgen and innate immune parameters (a–d), when participants’ age and body mass index (BMI) were controlled.

Innate immunity parameters				
	Model no 1	Model no 2	Model no 3	Model no 4
**a)**	***complement activity (ng/ml)***			
fT	−0.022 (0.84)			
*T*		0.156 (0.14)		
*DHEA*			0.146 (0.18)	
*DHT*				0.162 (0.14)
*Age*	0.038 (0.75)	0.082 (0.47)	0.096 (0.41)	0.110 (0.36)
*BMI*	0.054 (0.64)	0.035 (0.75)	0.029 (0.79)	0.014 (0.90)
*adjusted Model R* ^2^	<0.001	<0.001	<0.001	<0.001
*Model p*	<0.89	<0.42	<0.49	<0.43
**b)**	***lysozyme activity***			
fT	0.113 (0.30)			
*T*		0.020 (0.85)		
*DHEA*			0.161 (0.13)	
*DHT*				0.070 (0.52)
*Age*	−0.050 (0.67)	−0.087 (0.44)	−0.037 (0.75)	−0.064 (0.59)
*BMI*	−0.138 (0.22)	−0.119 (0.29)	−0.140 (0.21)	−0.133 (0.24)
*Adjusted Model R* ^2^	0.011	<0.001	0.023	0.004
*Model p*	<0.26	<0.40	<0.16	<0.34
**c)**	***phagocytic uptake***			
fT	−0.202 (0.06) −0.128 (0.25)*			
*T*		0.018 (0.86) −0.096 (0.37)*		
*DHEA*			−0.164 (0.13) −0.173 (0.11)*	
*DHT*				−0.176 (0.11) −0.200 (0.07)*
*Age*	−0.063 (0.59) −0.143 (0.24)*	0.017 (0.88) −0.122 (0.30)*	−0.043 (0.71) −0.150 (0.20)*	−0.056 (0.64) −0.172 (0.15)*
*BMI*	0.105 (0.35) 0.173 (0.14)*	0.066 (0.56) 0.161 (0.16)*	0.091 (0.41) 0.172 (0.13)*	0.107 (0.34) 0.195 (0.09)*
*Adjusted Model R* ^2^	0.010 0.002*	<0.001 <0.001	<0.001 0.016*	0.001 0.023*
*Model p*	<0.26 <0.37*	<0.91 <0.45*	<0.42 <0.22*	<0.38 <0.16*
**d)**	***ROS production***			
fT	0.061 (0.57) 0.147 (0.19)*			
*T*		0.001 (0.99) 0.067 (0.53)*		
*DHEA*			0.072 (0.50) 0.035 (0.75)*	
*DHT*				0.198 (0.07) 0.179 (0.11)*
*Age*	−0.029 (0.81) 0.039 (0.75)*	−0.052 (0.65) 0.003 (0.98)*	−0.027 (0.81) 0.006 (0.96)*	0.026 (0.82) 0.052 (0.67)*
*BMI*	0.169 (0.14) 0.069 (0.55)*	0.180 (0.11) 0.094 (0.41)*	0.169 (0.13) 0.095 (0.41)*	0.136 (0.23) 0.061 (0.60)*
*Adjusted Model R* ^2^	<0.001 <0.001*	<0.001 <0.001*	0.001 <0.001*	0.031 0.005*
*Model p*	<0.40 <0.46*	<0.45 <0.75*	<0.38 <0.82*	<0.11 <0.33*

*Standardized beta coefficients with *p* values in parentheses for analyses without statistical outliers for immune factors (N = 93 for phagocytic uptake, and N = 94 for reactive oxygen species [ROS] production).

Entries are standardized beta coefficients with *p* values in parentheses.

### The relationship between adaptive immune functions and androgens

Among the adaptive immune parameters, only the strength of the influenza post-vaccination response was positively predicted by fT (*p* = 0.03) and marginally significantly (*p* = 0.06) by DHT. Other immune functions were not correlated with any of the studied hormones (Table 4).

**Table 4:** Multiple regression models showing the relationship between each androgen and adaptive immune parameters (a–g), when participants’ age and body mass index (BMI) were controlled.

Adaptive immunity parameters				
	Model no 1	Model no 2	Model no 3	Model no 4
**a)**	***IgA levels (g/L)***			
fT	0.125 (0.24)			
*T*		0.010 (0.92)		
*DHEA*			0.055 (0.61)	
*DHT*				0.079 (0.47)
*Age*	**0**.**267 (0**.**02)**	**0**.**223 (0**.**05)**	**0**.**240 (0**.**04)**	**0**.**251 (0**.**03)**
*BMI*	−0.010 (0.92)	0.011 (0.91)	0.005 (0.97)	−0.005 (0.96)
*Adjusted Model R* ^2^	0.03	0.02	0.02	0.02
*Model p*	<0.10	<0.18	<0.16	<0.14
**b)**	***IgG levels(g/L)***			
fT	0.051 (0.64)			
*T*		0.118 (0.26)		
*DHEA*			0.004 (0.96)	
*DHT*				0.184 (0.09)
*Age*	−0.114 (0.33)	−0.106 (0.34)	−0.132 (0.26)	−0.061 (0.60)
*BMI*	−0.101 (0.37)	−0.102 (0.35)	−0.092 (0.41)	−0.132 (0.24)
*Adjusted Model R* ^2^	0.007	0.018	0.004	0.035
*Model p*	<0.30	<0.19	<0.33	<0.10
**c)**	***T lymphocyte (cells/µl)***.			
fT	−0.009 (0.93)			
*T*		−0.065 (0.53)		
*DHEA*			0.027 (0.80)	
*DHT*				−0.047 (0.66)
*Age*	**−0**.**288 (0**.**01)**	**−0**.**300 (0**.**08)**	**−0**.**275 (0**.**02)**	**−0**.**303 (0**.**01)**
*BMI*	0.051 (0.64)	0.056 (0.61)	0.046 (0.67)	0.060 (0.59)
*Adjusted Model R* ^2^	0.042	0.047	0.043	0.045
*Model p*	<0.07	<0.06	<0.07	<0.06
**d)**	***B lymphocyte (cells/µl)***.			
fT	−0.160 (0.14)			
*T*		−0.140 (0.18)		
*DHEA*			−0.051 (0.63)	
*DHT*				−0.077 (. 48)
*Age*	**−0**.**239 (0**.**04)**	−0.212 (0.06)	−0.197 (0.09)	−0.210 (0.08)
*BMI*	0.109 (0.33)	0.094 (0.40)	0.087 (0.43)	0.097 (0.39)
*Adjusted Model R* ^2^	0.019	0.015	<0.001	0.001
*Model p*	<0.19	<0.22	<0.42	<0.37
**e)**	***strength of flu post-vaccination response***			
fT	**0**.**231 (0**.**03)**			
*T*		0.001 (0.99)		
*DHEA*			0.080 (0.44)	
*DHT*				0.197 (0.06)
*Age*	−0.179 (0.11)	**−0**.**265 (0**.**02)**	**−0**.**238 (0**.**04)**	−0.183 (0.10)
*BMI*	−0.077 (0.47)	−0.035 (0.74)	−0.046 (0.67)	−0.079 (0.47)
*Adjusted Model R* ^2^	0.098	0.049	0.055	0.084
*Model p*	<0.005	<0.05	<0.04	<0.01
**f)**	***Proliferation rate after Con A stimulation (N*** = ***6*2*)***			
fT	0.018 (0.90)			
*T*		−0.054 (0.69)		
*DHEA*			−0.081 (0.55)	
*DHT*				−0.030 (0.82)
*Age*	−0.195 (0.20)	−0.219 (0.14)	−0.229 (0.12)	−0.212 (0.16)
*BMI*	0.106 (0.46)	0.118 (0.41)	0.124 (0.38)	0.116 (0.42)
*Adjusted Model R* ^2^	<0.001	<0.001	<0.001	<0.001
*Model p*	<0.55	<0.52	<0.48	<0.54
**g)**	***Proliferation rate after PWM stimulation (N*** = ***62)***			
fT	0.019 (0.89)			
*T*		−0.154 (0.26)		
*DHEA*			−0.134 (0.33)	
*DHT*				−0.016 (0.91)
*Age*	−0.145 (0.34)	−0.203 (0.17)	−0.198 (0.18)	−0.158 (0.29)
*BMI*	0.069 (0.63)	0.100 (0.48)	0.098 (0.49)	0.076 (0.60)
*Adjusted Model R* ^*2*^	<0.001	<0.001	<0.001	<0.001
*Model p*	<0.75	<0.48	<0.54	<0.76

Entries are standardized beta coefficients with *p* values in parentheses.

According to the hypothesis that interaction between testosterone and immune function may be mediated by stress hormones^20,40^ we repeated the analyses presented in Table 3 and Table 4 using cortisol as another predictor. The analyses show that none of the androgen-immune interactions changed after the adjustment for cortisol levels.

The immune response to influenza vaccination can be also analyzed according to the immunological standards, i.e. using a dichotomous scale (negative or positive immune response). A positive response is reflected by at least a four-fold increase in the antibody titer after vaccination^41^. To determine whether the group with a positive immune response (seroconverted) differed in any androgen levels from the group with negative immune response (lack of seroconversion), we compared androgen levels using the Mann-Whitney U test. The seroconverted group (N = 73) appeared to have on average higher fT levels than the group without seroconversion (N = 24) (Z = 2.32; *p* < 0.02). The serum concentrations of other androgens did not differ between these two groups.

## Discussion

Immune system efficacy is a very important element of human physiology, and is perceived as one of the best measures of an organism’s biological condition. Any defect in the immune function leads to inadequate protection and poses a significant risk to survival. According to the ICHH, morphological traits that are sexually dimorphic or related to sexual ornamentation may signal biological quality, and therefore could also reflect good immune quality. To test the hypothesis that androgen-dependent male ornamentation is also an honest signal of biological quality, we analyzed various parameters of immunity in relationship to different androgen levels in healthy adult males. In the well-nourished Western society studied here, we found very limited evidence for a relationship between some androgens and adaptive immunity. Our results do not confirm the hypothesis that androgens exert immunosuppressive effect. Below we discuss our results in details, categorized by the immune topic.

When controlling for age and BMI, none of the measured innate immune parameters were related to androgen concentrations. Our results are in contrast to the *in vitro* study by Marin *et al*.^42^, which documented an inhibitory effect of testosterone on neutrophil function. The experimental studies on mice with knockout of androgen receptors show, however, that these mice have functional defects of neutrophils including the lower production of proinflammatory cytokines, but retain the normal capacity of phagocytosis and ROS production^43^. We obtained the similar result in men i.e. no effect of androgens on neutrophils functions.

Our study corroborates the correlational study of Prall and Muhlenbien^22^ on complement activity and saliva DHEA concentrations. Although the data documenting a potential down-regulatory role of androgens in innate immunity are very limited, the evidence from correlational studies indicates that innate immune factors are independent of serum androgen concentrations.

We found that when controlling for age and BMI, androgens were not associated with the total number of T and B lymphocytes, i.e. major cells involved in adaptive cell-mediated and humoral immunity, respectively. A functional test of lymphocytes showed that androgen concentrations were not related either to lymphocyte proliferation after mitogenic stimulation or to total IgA and IgG immunoglobulin levels. Interestingly, the strength of the immune response to the influenza vaccine was positively predicted by the level of fT and marginally significantly by DHT. This indicates that hormones can affect rather the function of lymphocytes (in response to a stimulus) than their total number in blood.

The majority of human and animal *in vitro* studies which focused on quantitative analyses of lymphocytes have shown a negative impact of testosterone on lymphocyte proliferation^44–46^ or antibody production^47^. A limited experimental human study conducted on transsexual Caucasian women found no effect of testosterone administration on IgG levels^48^. The results of correlational studies, however, are inconclusive. Whereas some reported results similar to ours, i.e. observing no association between immunoglobulins and testosterone levels^49^, others found a positive relationship^50^. It is worth noting that in contrast to the study on Filipino men^51^, our study, as well as those of van Anders^49^ and Giltay *et al*.^48^ (the latter only on women) were conducted in well-nourished, affluent societies. These differences may suggest the influence of ecological factors on immune-testosterone interaction. In a correlational study linking lymphocyte function with androgens other than testosterone, Prall and Muehlenbien^22^ found that saliva DHEA level was negatively correlated with lymphocyte proliferative response. Because of the small sample size (only 20 men) and no controlling for participant age, adiposity, or health status, the conclusions of Prall and Muehlenbien should be, however, treated with caution.

In general, our results on the baseline immune parameters and androgens are compatible with the meta-analysis findings on many species by Foo *et al*.^51^. They showed that in contrast to experimental studies, there was no significant overall correlation between testosterone and immune function in correlational studies.

There are also inconclusive data for the relationship between androgen concentrations and the ability to produce specific antibodies post-vaccination. Our results show that men with higher levels of fT produce more specific anti-influenza antibodies in response vaccination. This finding is similar to those obtained by Rantala *et al*.^20^, who found a positive relationship between total testosterone levels and immune response to hepatitis B vaccination. The opposite finding for fT and response to influenza vaccination was reported by Furman *et al*.^21^. Their men sample, however, was small (N = 34), and the median age of participants was 63 years. Age is an important factor in immune response to vaccination it is because the response tends to decrease with age. In Furman *et al*.^21^, only 10 out of 37 men (27%) responded positively to the influenza vaccine by showing seroconversion. In our study 75.3% of the vaccinated men were seroconverted, what is similar to the rate obtained in healthy young people in another study (68%–84%)^52^. Furthermore, Furman *et al*.^21^ did not control for adiposity, which may influence the immune response to the influenza vaccine^53^.

Due to the small sample of men who were also vaccinated against tetanus in our study, we did not present the response analyses for this antigen. It is worth noting, however, that although nonsignificant (*p* = 0.11), the relationship between fT and the response to tetanus vaccination was in the same direction (R = 0.27) as that of the influenza vaccine.

There are a few possible explanations of higher immune reactivity in more androgenic men (with higher level of fT). Firstly, it is possible that in energetically replete populations (as in the case of our study), men with high genetic quality can afford to invest in more costly adaptive immune arm and at the same time in high androgens production. Such explanation agrees with the hypothesis that only individuals with high immune quality are able to incur the immunosuppressive effect of testosterone. Secondly, the proportional marginal viability cost that is associated with high testosterone level is lower for individuals with higher immune quality. According to Getty *et al*.^54^ the experimental and correlational studies might only reflect absolute cost, whereas it is proportional marginal fitness cost (i.e. in relationship to benefits) that might be crucial for an individual ability to develop sexual signal. Thirdly, because the immune activation in response to infection is related to down-regulation of testosterone levels, higher testosterone in men can be consider as a signal of better physiological condition (including immunity)^55^. Finally, if testosterone is not absolutely immunosuppressive (but rather immunomodulatory), high genetic quality individuals can invest more in maintenance both in high level of viability (reflected by immune efficiency) and in fecundity (active form of androgens).

Our results also indicate that to test the hormonal basis of the immunocompetence hypothesis, the immunological challenge (such as vaccination) may be more informative than baseline immune parameters or functional tests performed on isolated immune cells (such as neutrophil phagocytic uptake or lymphocyte proliferative response). It is because of the nature of immune system network function, when the basal levels of immune parameters occurring in healthy individuals do not necessarily reflect reactivity of immune cells in response to a real infection.

Overall, we did not confirm the ICHH as postulated by Folstad and Karter^2^. There is, however, one problem with this hypothesis. The positive, negative, or lack of relationship between androgens and immune function do not need to undermine the ICHH^54^. In the case of a positive relationship, one can assume that individuals with high testosterone levels can also afford high immunity, and in the case of a negative relationship, that healthy men are trading off between both. The majority of research validating the ICHH has been performed on birds, but results of animal studies are also difficult to interpret because of a lack of “normal range” definition for immunological parameters in healthy individuals. Higher levels of measured leukocytes or immunoglobulins may be interpreted as a higher immune potential (better immunity) or as a result of outgoing infection (weaker immunity)^6^. In the case of individuals living in Western societies, recent data do not support either the ICHH or the positive relationship between the traits perceived as attractive and immune functioning. Foo *et al*.^56^ for instance, found no relationship between components of men’s facial attractiveness and selected innate immunity factors. This indicates that testosterone-dependent facial masculinity does not have to signal the immune effectiveness.

In the light of previous results, the main question is why androgens exert different effects on immune cells *in vitro* and *in vivo*. Possibilities include molecular mechanisms responsible for local androgen conversion by immune cells, synthesis of specific enzymes, or patterns of sex hormones receptors expressed on immune cells. Firstly, it is possible that *in vivo*, testosterone can exert indirect effects on immune cells after local conversion by immune cells to estrogenic metabolites and binding to estrogen receptors^57,58^. It is well-known that estrogens up-regulate immune functions, and this may be one reason for the lack of immunosuppressive effect of testosterone observed in our study. Secondly, ARs are expressed by various immune cells and play an essential role in androgen-associated immune regulation (see^15^). Studies on mice lacking androgen receptors^59^ or experimental human studies^60^ have shown that the comprehensive impact of hormones on immune response depends not only on hormone concentrations but also on qualitative and quantitative expression profiles of specific immune-cell receptors present in various tissues. This evidence suggests that individual differences in the expression of ARs on various immune cells can explain why the same androgen levels may exert effects of varying magnitude on immunity. Lastly, the effect of AR signaling differs *in vitro* and *in vivo*. Yang *et al*.^61^ for instance, showed that AR activation exerted an inhibitory effect on prostatic epithelial cell proliferation *in vitro*, whereas *in vivo*, the activation had a stimulatory effect. According to above evidence, to better understand androgen-immune interaction, further investigation of ICHH assumptions should include not only hormone levels but also specific receptor expression patterns and enzyme synthesis by immune cells which convert androgens into other metabolites.

The second problem is related to different physiological or ecological factors influencing immune-androgen interaction. Both animal and human studies indicate that immunity development in early life can be crucial. In this respect, McDade *et al*.^24^ suggests three potential elements: “(a) the availability of nutritional resources, (b) the intensity of pathogen exposure and (c) signals of extrinsic mortality risk”. The combination of these factors may lead to distinct immunomodulatory effects by androgens. There are reports showing trade-offs between different fitness components when resources are limited^24,62^. Some animal studies showed that food availability can influence the relationship between testosterone levels and immunity^63^ and that leptin may prevent the immunosuppressive role of testosterone^64^. Other factors which may affect testosterone-immune interactions are stress^20^ and living conditions^65^. Trumble *et al*.^65^ found that cell-mediated response to mitogens was inversely related to testosterone levels in Tsimane men aged 40–89 from an energy-limited subsistence population, which faces a high pathogen burden and is therefore immunologically stressed.

Finally, it should be noted that the majority of empirical studies (both experimental and correlational) are mainly designed to measure absolute cost of “signals” (androgens level) which will might be not sufficient to define viability-fertility (i.e. immune-hormones) trade-offs^54^. According to Getty *et al*.^54^ costly signals should be considered as an investment instead of handicaps, and we should avoid the assumptions that individuals who express higher sexual signals must have “wasted” more viability to develop them than lower quality individuals with lower expression of sexual signals. The ICHH prediction is based on an absolute cost associated with the levels of expression sexual signals, whereas it is the marginal fitness cost and benefits (not easily measurable in empirical studies) that might be crucial^54^.

Despite some limitations, the strength of our study is derived from four factors: (a) A relatively large sample of men in their reproductive age, which is important when testing evolutionary hypotheses; (b) a variety of immune biomarkers evaluated; (c) four different androgens studied; (d) controlling for general health status using medical biomarkers.

It is also worth noting that although our study is mainly correlational, it is the first study testing the relationship between different androgens concentration and many other immune functions simultaneously. This means that in contrast to previous studies, our conclusions are about the general immune-androgens *in vivo* interactions that are based on multiple immune parameters.

In summary, we found that fT and DHT are probably the hormones most relevant for studying immune parameters potentially affected by androgens. These two reactive forms of androgens positively influence adaptive immunity, i.e. the strength of the response to the influenza vaccine, and appear to have no effect on humoral or cell-mediated innate immunity. This finding contrasts with the immunosuppressive function of androgens observed *in vivo*. The relationship between androgens and immune function is, however, complex, and probably restricted only to particular immune parameters and does not apply to all. Therefore we suggest treating androgens as immunomodulators rather than immunosuppressants, as supported by the important role of ecological factors in moderating the relationship between immunity and androgens. Further investigations regarding the association between serum androgen concentrations, ARs, and various immune functions are required.

## Methods

### Participants

One hundred and thirty-four men (aged 18.9–36.7 years) who were volunteers and declared no health problems were recruited for the study. Participants who did not complete the study, reported an infection between the two office visits, had above-normal ranges in any blood parameters, had body mass index (BMI) or body fat values above or below the 3^rd^ standard deviation, and were outliers in sex hormone levels, were excluded from the analyses. The final sample was 97 healthy men (aged 19–36.7 years, mean age 27.2). Thirty-five of these men were also tetanus-vaccinated (aged 20.7–36.7; mean age 28.7) and evaluated for immune response to tetanus toxoid. The lymphocyte T proliferation rate was assessed for 62 participants. Most of the participants lived in Wroclaw (Poland) and the suburbs (85%), had university level education (63%) or be students (24%).

### Patient examination scheme

Participants examination was performed in local private clinic. The general study procedure for recruited participants consisted of two visits spaced 4 weeks apart. Both visits were scheduled in the morning hours. The first visit included medical qualification to vaccination, blood sample collection for biochemical, immunological, and hormonal analyses, influenza or both influenza and tetanus vaccine injection, and height, weight, and body fat percentage measurements. Four weeks later, during the second visit, participants submitted to blood sample collection to evaluate the strength of the immune response to vaccination and filled in questionnaires on illnesses after the first visit. All participants were tested for blood morphology and levels of fasting glucose, creatinine, alanine aminotrasferase, aspartate aminotransferase, and C-reactive protein to evaluate general health status. All participants were vaccinated against seasonal influenza using Vaxigrip® (SanofiPasteur, S.A. Lyon, France), containing influenza strains recommended by the World Health Organization for 2013/2014 and 2014/2015 influenza season. Some participants were also vaccinated against tetanus toxoid using Tetana (Biomed S.A, Kraków, Poland).

Participant recruitment and vaccinations were performed during two influenza seasons, 2013/2014 and 2014/2015, from September to March. Blood samples for biochemical, immunological, and hormonal analyses were collected in the morning hours between 7:30 and 9:00.

### Immune function measurements

Participants’ immune system quality was determined using key immune mechanisms constituting both innate and adaptive immune responses.

It is important to note that we used immune functions representing various aspects of immune defense. We assessed the parameters: (1) creating humoral innate immunity (total complement and lysozyme activity); (2) constituting cellular innate immunity (the neutrophil function including phagocytosis and reactive oxygen species production) as well as those (3) involved in cell-mediated immunity (T lymphocyte count and proliferative response after mitogen stimulation), and (4) involved in humoral-mediated immunity (B lymphocyte counts, IgA, IgG and the immune response to influenza or tetanus vaccinations). All measured parameters are included in the test panel for evaluating the immune functioning and were recommended as a screening test for detect primary immunodeficiency^66^.

#### Innate immune function

Total complement activity was measured in zymosan-activated serum using commercial enzyme immunoassay kit (MicroVue, Quidel®). Test procedure was performed according to the manufacturer instruction. Results were expressed in ng/ml.

Lysozyme activity of human serum was measured using turbidimetric assay and *Micrococcus lysodeikticus* cell suspension, and expressed as a difference in absorbance value between control and test sample.

Phagocytic uptake by neutrophils was measured using commercial kits (PHAGOTEST, Glycotope®). The mean fluorescent intensity of phagocytosing neutrophils was calculated using WinMDI software.

Reactive oxygen species (ROS) production by isolated neutrophils after PMA stimulation, was measured using luminol-dependent chemiluminescence assay (CL). The results were expressed as area under chemiluminescence curve (AUC) for stimulated neutrophils divided by AUC for controls.

#### Adaptive immune function measurement

The absolute number of T cells and B cells in peripheral blood was measured with appropriate commercial TriTest (Beckton Dickinson®). The results were calculated using BD CellQuest software and expressed as the number of positive cells (CD3 or CD19) per microliter of blood (cells/µl).

The lymphocyte proliferative response after mitogen stimulation was measured using [3H] thymidyne incorporation assay (modified method described in). Results were expressed as stimulation index (SI) calculated as count per minute (CPM) for mitogen-stimulated sample divided by CPM for unstimulated controls.

Total serum immunoglobulin IgA and IgG levels were measured with enzyme-linked immunosorbent assay, using previously calibrated reagents concentration and series of participant’s serum samples dilution. Results were expressed in g/L.

The strength of immune response to flu vaccine the titre of specific IgG antibody was measured before and 4 weeks after vaccination^41,52^. Anti-flu antibody titre was measured with the standard ELISA method, using microplates coated with influenza vaccine antigens (Vaxigrip®, SanofiPasteur) and previously calibrated reagents and participant’s serum sample dilution. Immune response to vaccine was expressed as fold-increase in specific antibody titre from pre- to post-vaccination.

The strength of anti-tetanus IgG was measured using commercial kits (DEMEDITEC®), and previously calibrated participant’s serum sample dilution. Test procedure was performed according to the manufacturer instruction. The results were expressed as fold-increase between pre- to post-vaccination specific antibody concentration.

The immunological methods and test procedure are described in details in the supplementary materials (SI).

### Hormonal analysis

Total testosterone, fT, DHEA, and DHT concentrations were measured in serum samples. Measurements were conducted by enzyme-linked immunosorbent assay (ELISA) and appropriate commercial kits (DEMEDITEC®). Serum sample dilutions and test procedure were performed according to the manufacturer’s instructions. The intra- and inter-assay reproducibility and assay sensitivity were, respectively: <4.16%, <9.94%, 0.083 ng/ml (for T); <10%, <10%, 0.06 pg/ml (for fT); <7.9%, <6.9%, 0.07 ng/ml (for DHEA); <6.25%, <7.47%, 2.23 pg/ml (for DHT). Hormonal concentrations were calculated in relation to the standard curve and expressed in ng/ml for T and DHEA and in pg/ml for fT and DHT.

### Statistical analysis

Normality for variables was assessed with Shapiro-Wilk tests. Despite logarithmic transformation, the majority of variables were still not normally distributed. A series of Spearman correlations using raw data were carried out to test inter-correlation between immune parameters and the relationship between immune functions and participants’ age, BMI, percent of body fat, and each hormone. The relationship between immunity and hormones was also tested with different multiple regression models, controlling for known immunomodulatory factors such as participants’ age and BMI. Regression models were constructed following natural logarithmic transformation of data (except age) to normalize the residuals distribution. The mean androgen levels between the seroconverted group and group without seroconversion were compared using Mann-Whitney U test. Analyses were carried out using Statistica 12 (StatSoft Inc.).

### Ethics

The research was approved by the Bioethics Commission at the Lower Silesian Chamber of Physicians and Dentists’ ethics committee (2/PB/2013). All participants read and signed the informed consent form.

All medical procedures, including participants examination, blood collection and vaccination have been conducted by certified medical staff in the private clinic. All procedure was consistent with the guideline included in the “Declaration of Helsinki – Ethical Principles for Medical Research Involving Human Subjects” formulated by World Medical Association in 2013 (https://www.wma.net/policies-post/wma-declaration-of-helsinki-ethical-principles-for-medical-research-involving-human-subjects/).

### Data availability

The authors declare that the data supporting the findings of this study are available in supplementary information files.

## Electronic supplementary material

Immunological methods

Dataset 1
