# Supplementary material for: No evidence for the immunocompetence handicap hypothesis in male humans
Source: Sci Rep. 2018 May 9;8:7392. doi: 10.1038/s41598-018-25694-0 (PMC5943526; doi:10.1038/s41598-018-25694-0)
Supplement: Supplementary file 1 — Immunological methods [file 41598_2018_25694_MOESM1_ESM.doc]

**TITLE: No evidence for the immunocompetence handicap hypothesis in male humans**

***Nowak J.^1,$^,*, Pawłowski B.^1,$^,*** *Borkowska B. ^1^, Augustyniak D. ^2^ Drulis-Kawa Z. ^2^*

SUPPLEMENTARY INFORMATION (SI)

**Assessment of immunological parameters**

***Innate immune parameters***

*1. Total complement activity*

The activation of all three complement pathways based on the membrane attack complex formation and detected by the presence of C5b-C9 was assayed in zymosan-activated serum using commercial EIA kits (MicroVue, Quidel®, USA) according to manufacturer's instructions. Test procedure of serum activation was described previously [67]. Absorbance was measured using spectrophotometer Asys UVM340 (Biochrom®), results were calculated in relation to standard curve and expressed in ng/ml.

*2. Lysozyme activity*

Lysozyme activity of human serum was measured by the lysis assay using *Micrococcus lysodeikticus* (Sigma Aldrich®) cell suspension. 15mg of frozen *Micrococcus lysodeikticus* was suspended into 20ml phosphate buffer (pH 6.2) (0.075% bacteria suspension). 175µl of suspended bacteria were added to microplate wells (Maxisorp, NUNC™) containing 25µl of serum (test sample) or buffor (control), both test and control were assayed in duplicate and the values were averaged. The decrease in the turbidity of the test sample and control sample was measured after 20 min. incubation using λ=450 nm and spectrophotometer Asys UVM340 (Biochrom®). The results were calculated as the difference in absorbance value between control and test sample. The greater difference reflected higher lysozyme activity in a participant's serum.

*3. Neutrophil phagocytic activity*

*a) phagocytic uptake*

Phagocytic activity of peripheral blood neutrophils (PMNs) was assayed using functional response such as phagocytic uptake and generation of reactive oxygen species (ROS). The phagocytic uptake was performed using commercial kits (PHAGOTEST, Glycotope®, Germany) according to manufacturer's instructions. Fluorescence of FITC labeled *Escherichia coli* ingested by granulocytes (FITC positive granulocytes) was measured by FACs Calibur flow cytometer (Becton Dickinson®). To quantify microbial uptake, WinMDI 2.9 software was used. The results were expressed as mean fluorescent intensity of phagocytosing neutrophils

b) reactive oxygen species production

The generation of ROS production, defined as oxidative burst by isolated PMNs, was measured using luminol-dependent chemiluminescence assay (CL) in white flat-bottom microplates (NUNC®, Denmark), as described previously with slight modifications [68]. PMNs were freshly isolated by density gradient separation method using Percoll®. Briefly, PMNs (10^5^cells/well) were stimulated with PMA (Phorbol 12-myristate 13-acetate) at final concentration 0.1µM in the presence of 50µM luminol as luminescent probe. The CL kinetics was measured using luminometer for 45 minutes and recorded as relative light units (RLU). Each sample was run in triplicate and the values were averaged. The area under chemiluminescence curve (AUC_CL_) for PMA-stimulated neutrophil chemiluminescence (AUC-PMA) and for baseline luminol chemiluminescence (AUC-control) were calculated. Relative CL was calculated as mean AUC_CL_- PMA divided by AUC_CL_-control. Results were expressed as a mean ± SD.

**Adaptive immune parameters**

*1. The absolute count of T and B lymphocytes*

The quantitative assessment of the key population of peripheral blood lymphocytes (T cells and B cells) was carried out using fluorochrom-labeled monoclonal antibodies and measured on FACs Calibur flow cytometer (Becton Dickinson®). Commercial kits from Becton Dickinson® were used to identify and determine absolute counts of mature human B lymphocytes (CD19): *TriTest CD3 FITC/CD19 PE/CD4 PerCP* and T lymphocytes (CD3): *TriTest CD4 FITC/CD8 PE/CD3 PerCP.* The specimen collection and preparation, as well as test procedure was performed according to manufacturer's instructions. The results were calculated using BD CellQuest software and expressed as the number of positive cells (CD3 or CD19) per microliter of blood (cells/µl).

*2. The functional assays of lymphocytes:*

*a) The proliferative response after mitogen stimulation*

The lymphocyte proliferation test was performed using [3H] thymidyne incorporation assay (described previously with modification in 69). Isolated PBMC were suspended in medium supplemented with 20% autologous plasma and added in triplicate to microculture plates wells (Thermo Fischer®, USA) at final concentration 1x10^5^cells/well. Unstimulated lymphocytes (control wells with medium), stimulated with 20µg/ml concanavalin (Con A stimulated lymphocytes) and with 0.1 µg/ml pokeweed mitogen (PWM stimulated lymphocytes) were incubated for 72 hours in 37^0^C with 5% CO_2_. 20µl [3H] thymidyne was added to each well 16 hours prior to harvesting. After termination by saponin, samples were transported into Whatman® filter circles, dried and rinsed successively in 10% trichloroacetic (for 30min, 2-8^0^C), 60%, 80%, 96% ethanol and eter. Dried samples were placed into separate tube containing Ultima gold liquide (Perkin Elmer®, USA) and then were measured using Beckman Coulter. The measure was expressed as count per minute (cpm). Results were expressed as stimulation index (SI) calculated as CPM for mitogen-stimulated sample divided by CPM for unstimulated controls.

*b) Total IgA and IgG levels*

Serum immunoglobulin concentrations were measured with enzyme-linked immunosorbent assay (ELISA) using previously calibrated reagents concentration and series of participant's serum samples dilution [according to methods describes in 70]. Microplates (Maxisorp, NUNC®, Denmark) were previously coated with polyclonal rabbit anti-human IgA or IgG (DAKO®) at final concentration 3 µg/ml. Serum samples were diluted: 20 000x, 40 000x, 80 000x, 160 000x for IgA and 80 000x, 160 000x, 320 000x, 640 000x for IgG. Enzyme conjugated antibodies (HRP labelled rabbit anti-human IgA or IgG) (Dako®, Denmark) were diluted 6 000x for IgA and 10 000x for IgG. After series of incubations with blocker, participant's serum sample dilutions, enzyme-conjugated antibodies, and substrate, the absorbance was measured using λ=490 nm on spectrophotometer Asys340. Results were calculated in relation to the standard curve and expressed in g/L.

*c) Anti-flu antibody titre detection:*

The titre of specific anti-flu IgG antibody was measured before and 4 weeks after vaccination [41, 52]. Anti-flu antibody titre was measured with the standard ELISA method [71]. Briefly, microplate (Maxisorp, NUNC™, Denmark) was coated with 0.1µg/ml influenza vaccine antigens (Vaxigrip®, SanofiPasteur, France). Pre-vaccination serum samples were mostly diluted 20 000x, 40 000x, 80 000x and 160 000x whereas post-vaccination samples were diluted 40 000x, 80 000x, 160 000x, 320 000x and 640 000x. After series of incubations with blocker, participant's serum sample dilutions, enzyme-conjugated antibodies, and substrate, the absorbance was measured using Asys UVM (Biochrom®) and λ=490 nm. The antibody titre was defined as the reciprocal value of the highest dilution of serum sample giving a positive test reaction below OD=1. Since all participants had some level of specific anti-flu antibody in blood sample before flu shot, the strength of immune response to vaccine was expressed as fold-increase between pre- to post-vaccination specific antibody titre. The immune response to flu vaccine was analyzed as continuous variable as well as categorized variable. Positive response to flu vaccine (seroconversion) was defined as minimum 4-fold increase in antibody titre, whereas lack of response (lack of seroconversion) was defined as lower than 4-fold increase [41, 52]. In the latter group characterized by positive response (seroconversion) was compared to the group with the lack of response to vaccination (lack of seroconversion).

*d) Anti-tetanus* *antibody titre detection:*

The evaluation of specific anti-tetanus antibody was performed before and 4 weeks after vaccination, using commercial kits (DEMEDITEC®, Germany). Serum samples dilution and test procedure were conducted according to manufacturer instruction supplied with the kit. Pre-vaccination serum samples were mostly diluted 1x, 2x whereas post-vaccination samples were diluted 2x, 4x, 8x. The results were calculated in relation to standard curve and expressed in IU/ml. All participants had detectable level of specific anti-tetanus toxoid antibody in blood sample before vaccination. The strength of immune response to tetanus vaccine was expressed as fold-increase between pre- to post-vaccination specific antibody concentration. Due to small sample size, the immune response to tetanus vaccine was analysed only as a continuous variable.
